# Round the Hospitals

**Published:** 1917-12-29

**Authors:** 


### ROUND THE HOSPITALS.

WORKERS in hospitals of every description throughout the country have good reason to be grateful that their field of labour has been, by the continuous, inspiriting, and self-denying personal service of their predecessors, cultivated upon principles which rest upon an ideal Christmas for every inmate of a British hospital. This personal service has, in fact, produced an outpouring of gratitude and an awakening of spirit that have proved of infinite value to individual patients and to the nation and people as a whole. We have said enough in recent issues to make the grounds of this conclusion fully apparent, but there is one additional link between hospital workers and the staff of THE HOSPITAL this Christmas which must make them akin, for the reason that Christmas Day falls upon a Tuesday, and so brings with it many added difficulties, for the working days of the week are thus

**ROUND THE HOSPITALS**—*(continued)*.

absorbed in advance by the demands of Christmas upon every worker concerned. It means to them that they must have continuously worked without intermission under a heavy weight of responsibility for ten consecutive days at least, owing to the necessity of crowding twelve days' work into eight days or less. For the weekly journalist it will prove impossible to describe this year, in the press, the Christmas festivities and rejoicings in our hospitals with any fulness, if at all, until after a much longer interval than usual. The consolation of everybody interested should, however, be that we are all united in personal service for others, who, without our aid, must have had a war Christmas with its weight of care, unrelieved and burdensome.

How many readers, we wonder, remember fifty or even forty years back? To how many does memory bring to mind with clearness the continuous dreariness of the hospital in those earlier days, which was aggravated at Christmas by the white-washed walls, the absence of any real attempt at comfort as it is now understood, and the cruel conditions under which the nurses often had to sleep. When the awakening to better things came in the late 'sixties, the difficulties of getting started, of introducing changes and carrying through an altered system, full of cheer and brightness, were great indeed. Prejudice had to be overcome, public opinion had to be awakened, personal interest excited on the part of the younger inhabitants of the centres where each hospital was situated, and appeals to the hearts and heads of the community had to be conveyed to an ever-increasing number of individuals, most of whom had to be awakened and convinced that a Christmas festival in a hospital was possible and even desirable.

THE mortality in hospitals at that time, when serious operative interference took place, was appalling. One in three of the patients so treated in many of the larger hospitals died. The absence of comfort, the often white-washed walls, the unsuitable furniture, the quality of the service, the food arrangements, and the equipment generally, if described with accuracy and force, would hardly be credited. Let it suffice to remind our readers that residents outside a hospital, for the most part, regarded it as a place to be shunned by the wise, and the soundness of this view was supported by the site chosen for many hospitals, which did not aim at bringing these institutions under the continuous notice of the population which habitually used the leading thoroughfares. We will not depress our readers by attempting their description. We content ourselves by giving an illustration of a ward in the Middlesex Hospital of an even earlier period, taken from the "Microcosm of London," by Rowlandson and Pugin, published by R. Ackermann in 1808, which contains some hundred coloured illustrations of great merit and historical interest. We should be happy to show these to any of our readers who are interested in this long-ago feature of the metropolis of the Empire.

THIS illustration emphasises the truth that is often forgotten, that hospitals, like other institutions in each period of a nation's growth, fairly indicate a stage of development in regard to the life and surroundings of the people. This illustration (see opposite page) should do two things. It should bring to the mind of the beholder what a steep road had to be travelled by reformers and idealists before the days when social science became popular, and scientific and hygienic developments forced their way into public notice and became an ever-extending power in providing for the legitimate claims and well-being of all casses of the community. Let us all remember with gratitude that, some twenty years earlier than the period we have in mind, Florence Nightingale was learning to be a nurse, and in training for her life-work. Later she was followed by Parkes, the father of hygiene, and Lister, the great surgeon. The teaching and work of all these pioneers resulted in the enormous saving of life which makes our hospitals of to-day Palaces of Healing, essential to the sick of every class of the community. Hence the universal feeling properly is that, when grievously ill, or in need of surgical interference and adequate nursing in hygienic surroundings of the best, there is no place like a hospital, whatever the social position of the patient may happen to be. The sick nowadays owe continuous gratitude to the pioneers in hospital development and construction, in the enforcement of hygiene, the ever-growing efficiency of attendants on the sick, the classification of patients in quarters especially planned to further their restoration to health, and the increasing comfort and general welfare provided.

IT does us all good to look back sometimes, that we may be encouraged to go forward with increased energy and confidence. Those who wish to play the most useful part during their residence on this planet are fortunate if they have been taught in their youth to study biography. It is equally true that those who aspire to further progress in science and treatment, and to prove helpful guides to their fellows, should initially devote time and study to the history of the institutions and methods of past decades. They should further master the causes which have contributed to make the hospitals and kindred institutions in this country, when weighed by the knowledgeable from every point of view, institutions which are justly admitted to be amongst the best types the world contains.

THE promise as we go to press of genial weather on the 25th instant is not encouraging, but then the shortest day in the year is not yet behind us. We can only hope that the weather will be kinder than it proved to be in two of the last thirty years, when Christmas Day was concealed in fog that could be felt, and everything was practically hung up,

### ONCE A WARD IN MIDDLESEX HOSPITAL.

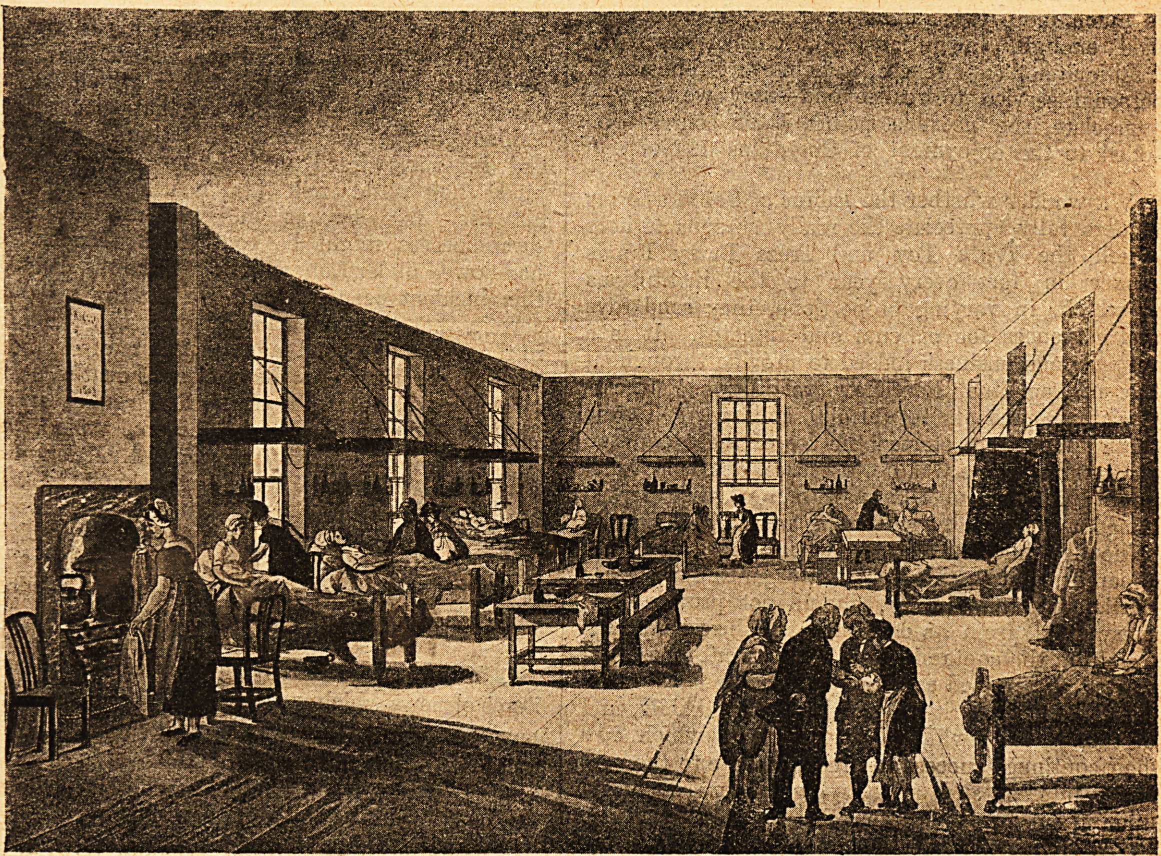

**A Drawing to Study with Experience.**

**ROUND THE HOSPITALS—(*continued*).**

including the inhabitants of London. We look forward to bright wards, merry faces, an atmosphere charged with merriment, thanksgiving, and song. We hope to meet some old friends who too seldom cross our path, owing to the continuous claim on the time of all who take, by necessity or choice, an active and serious part in the daily life of the community. Our hospital has been the centre of life and brightness on Christmas Day for at least thirty years. We have always had the tradition of meeting in the hospital on this day late and present members of the medical and nursing staff, present and former members of the visiting medical staff, governors who are devoted to the welfare of our institution, some old patients and friends who have come to see them, until we now feel that Christmas Day without a few hours in the hospital with the patients and staff would not be worth living. Then there are few sights and memories better worth treasuring than the Christmas Tree or Trees, and the amusement of the children in the out-patient department. Here are exhibited in characteristic fashion the habits and fancies, the charm, alertness, and vitality of the London child. Everybody has come to have a good time, and each takes care that he or she will have it.

THOSE of us who live outside the hospital often miss one of the most fascinating and charming incidents, that of the carol singers, whose voices cheer and give hope and expectation to the patients throughout the building. Tradition has led the singers to perambulate the corridors, which may add immeasurably to the charm of the carols and the winsome effect produced on the occupants of the beds. Father Christmas again! What a joy his personality gives to us elder ones who have seen many predecessors, have known many of them as friends, and who delight to study make-ups, compare notes, and then debate what in fact are the real and probable features of interest, encouragement, and cheer which Father Christmas *in propria persona* would present to the beholder! To many the service in the chapel is an event not to be missed and always to be thankful for. It gives real strength, proper perspective, and an uplifting outlook for the days to come. And this year we have our warriors with us, who, as an illustration in a recent number testified, are always bright, always smiling if you will, thoughtful for others, forgetful of themselves, and an asset of surpassing value, by example, to the whole hospital population. So, taking it all round, war-time has its charm and value, despite the special difficulties and added

**ROUND THE HOSPITALS—(*continued*).**

duties and responsibilities which it creates. There is much more that might be urged, but we have said enough, in anticipation, out of treasured traditions and experiences, to help every renderer of personal service to realise and—may we say?—appreciate, the possible points their contribution added to the enjoyment of Christmas 1917.

WE wonder whether the Editor of *Truth* and his readers fully appreciate the really seasonable help which the *Truth* Toy and Tress Barry Funds render in fact every year to the institutions to which, in varying proportions, they send toys and sixpences for patients and inmates. It is a very serious business indeed to provide throughout the metropolitan Poor-Law institutions suitable Christmas presents for 11,500 children. Before these funds were started the lot of these children at Christmas-time was not enviable, and it is a blot upon the political administration of the United Kingdom, especially when the war is making the value of child life to the whole community so apparent, that the Government organisation, under Parliamentary authority, should not provide that every child, from its earliest days, should be kept free from the contamination of the workhouse, and started and trained as a free citizen, under the best auspices, to make it free in thought, in habits, and in fact. Meanwhile the two funds, which owe their continued success in great measure to the personal interest and self-denying labours expended upon them by the present Editor of *Truth*, Mr. R. A. Bennett, render a service to humanity, and make the lot of these little ones, on one day in the year at any rate, something to be remembered with grateful recollection.

AND now let us have one good laugh together. How many readers will believe that within thirty years a great personage, of commanding authority with the people, one day stopped his carriage on London Bridge and altered the instructions to his coachman from: A hospital for which he was bound, to Home again? What caused this sudden change of purpose? Simply an inborn and inherited dread that, if he visited the hospital, he would risk his health and possibly his life, for it was in fact a place where dire diseases might be contracted. Much water has flowed under London Bridge since the day when this incident occurred, but we are by no means certain that this old tradition does not still linger in the minds of a good many members of the public, though experience to the contrary, on the part of millions of people, has changed the point of view until an English hospital has become a place to be wooed and won, rather than avoided and shunned. Anyway, we hospital people who know all about the hospitals can rejoice and be glad, that so many years of our lives have been spent in association with them, doing the highest kind of duty which it is the privilege of men and women to render to their fellows.

☛ IT is our invariable practice to pay for all items of news which we may publish. The minimum payment is 5s. Paragraphs of about twenty lines in length—that is to say, of 150 words or less—are preferred. Contributions should be addressed to The Editor, THE HOSPITAL, 28 and 29 Southampton Street, Strand, London, W.C. 2, and, to be in time for the current week's issue, should reach him by the first post on Mondays.

